# Males prefer virgin females, even if parasitized, in the terrestrial isopod *Armadillidium vulgare*

**DOI:** 10.1002/ece3.3858

**Published:** 2018-02-21

**Authors:** Margot Fortin, Catherine Debenest, Catherine Souty‐Grosset, Freddie‐Jeanne Richard

**Affiliations:** ^1^ Laboratoire Ecologie et Biologie des Interactions Equipe “Ecologie, Evolution, Symbiose” Université de Poitiers, UMR CNRS 7267 Poitiers France

**Keywords:** copulatory organ, mate choice, symbiosis, *Wolbachia*

## Abstract

In many species, males increase their reproductive success by choosing high‐quality females. In natural populations, they interact with both virgin and mated females, which can store sperm in their spermatheca. Therefore, males elaborate strategies to avoid sperm competition. In the terrestrial isopod *Armadillidium vulgare*, females can store sperm and produce several clutches. Moreover, this species can be parasitized by *Wolbachia*, which feminizes genetic males, transforming them into functional females. Our study compared attractiveness and mate choice when a male is exposed to both virgin and experienced females (i.e., females who have produced offspring and rested for 6 months), with or without *Wolbachia*. Our results revealed that males are more attracted to virgin females than experienced females, even if these virgin females are parasitized. Moreover, the chemical analysis highlighted different odors in females according to their reproductive and infection (*Wolbachia‐*free or vertically *Wolbachia*‐infected) status. Males attempted copulation more frequently and for longer with virgin females, even if *Wolbachia*‐infected, while experienced females refused further copulation. The evolutionary consequences of both male choice and female resistance on their fitness are discussed in this study.

## 1 INTRODUCTION

Mate choice is broadly defined as individuals’ preferences for members of the opposite sex leading to preferential mating (Andersson, 1994). Mate choice criteria are usually associated with evolutionary benefits in terms of fitness for the chooser, and their choice is made when these benefits outweigh the costs of being selective and wasting both energy and opportunities (Bonduriansky, 2001). Most studies looking at mate choice investigate the female choice because of their greater investment in reproduction (Bateman, 1948). However, an increasing number of studies have provided evidence that males can also be the choosy sex, in particular: (1) when the number of females available exceeds the males’ capacity to mate; (2) when there are variations in the quality of females (Edward & Chapman, 2011; Lihoreau, Zimmer, & Rivault, 2008). In both cases, males can increase their reproductive success by choosing high‐value females. The value of a female may depend on different factors such as their body size (e.g., in the hemiptera *Gerris lateralis*: Rowe & Arnqvist, 1996; in the gastropoda *Littoraria ardouiniana*: Ng & Williams, 2014; in the hemiptera *Anasa andresii*: Hamel, Nease, & Miller, 2015; in the salamander *Plethodon shermani*: Eddy et al., 2016), their age (e.g., in the onion fly *Delia antiqua*: Mcdonald & Borden, 1996; in *Drosophila hibisci*: Polak, Starmer, & Barker, 1998); and their infectious status (Beltran‐Bech & Richard, 2014). In addition, in natural population, males can interact with virgins or previously mated females. In the mouse *Mus musculus*, males are less likely to mate with recently mated females but, if they do choose them, they greatly increase their investment, by performing more intromission (e.g., bouts of penile insertion during each of which the male performs multiple intravaginal thrusts) before ejaculation and by enhancing copulation latency in order to override sperm competition (Ramm & Stockley, 2014).

In polyandrous species in which females can store sperm in their spermatheca, sperm competition has been found to be a factor of selection (Carazo, Sanchez, Font, & Desfilis, 2004; King, Saporito, Ellison, & Bratzke, 2005; Klein, Trillo, Costa, & Albo, 2014; Schneider, Zimmer, Gatz, & Sauerland, 2016; Scott, 1986). Moreover, several studies of polyandrous species have revealed a refractory period (time when females refuse new mating) between successive matings. The length of this period varies widely, from hours to days depending on the species (e.g., in the parasitoid wasp *Spalangia endius*: King et al., 2005; in the fruit fly *Batrocera cacuminatta* and *Batrocera Cucumis*: Chinajariyawong, Drew, Meats, Balagawi, & Vijaysegaran, 2010; in the psyllid *Bactericera cockerelli*: Guedot, Horton, Landolt, & Munyaneza, 2013; in the spider *Paratrechalea ornata*: Klein et al., 2014; and in *Batrocera dorsalis*: Wei et al., 2015). A refractory period between mating can be advantageous in terms of fitness, resulting in the female producing more offspring (Wei et al., 2015). A refractory period may also be caused by a negative consequence of multiple matings. Indeed, female behavioral resistance to mating might be due to injury caused by a first mating, as is the case for the dung fly *Sepsis cynipsea*, where the male genitalia harbor numerous spiny chitinous structures (Blanckenhorn et al., 2002). In species for which a refractory period occurs, female resistance is another factor that may influence mate choice and reproductive behavior.

In addition, infection status may also change mate choice in several species (Beltran‐Bech & Richard, 2014). The intracellular alpha proteobacteria *Wolbachia* are very widespread, occurring in insects, arachnids, nematodes, and crustaceans (Baldo, Prendini, Corthals, & Werren, 2007; Bouchon, Rigaud, & Juchault, 1998; Sironi et al., 1995; Werren, Windsor, & Guo, 1995). These vertically transmitted bacteria are highly manipulative symbionts which have different reproductive effects depending on their host, including cytoplasmic incompatibility, parthenogenetic induction, male killing, and feminization of genetic males (Werren, Baldo, & Clark, 2008). Moreover, the bacteria *Wolbachia* can influence mating preferences (Moreau, Bertin, Caubet, & Rigaud, 2001). In *Armadillidium vulgare*,* Wolbachia* induce the feminization of genetic males, transforming them into phenotypic and functional females (Juchault, Rigaud, & Mocquard, 1992; Martin, Juchault, & Legrand, 1973). The *Wolbachia* infection has negative effects on females’ learning and memory capacities (Templé & Richard, 2015), their survival (Braquart‐Varnier et al., 2008) and their attractiveness for mating (Moreau et al., 2001). Males perform more copulations and interactions and invest more sperm in *Wolbachia*‐free than in *Wolbachia*‐infected virgin females (Moreau et al., 2001).

We used the terrestrial isopod *A. vulgare* (Crustacea, Isopoda), as a model for male mate choice (Beauché & Richard, 2013; Moreau et al., 2001). Indeed, in this gregarious species, males can live in mixed groups and potentially interact with females with different life histories. Multiple matings occur in this species, and females are able to store sperm from previous matings and produce several clutches after mating (Howard, 1943; Johnson, 1976, 1982; Moreau, Seguin, Caubet, & Rigaud, 2002). *A. vulgare* males can discriminate between and show a preference toward females depending on their molting status from a short distance, mainly based on chemical cues (Beauché & Richard, 2013). Although the chemicals involved have not yet been fully identified, chemical extracts with dichloromethane have been found to contain attractive compounds (Beauché & Richard, 2013). After mating, females carry their offspring for 1 month in their marsupium before releasing them. The females may then perform another parturial molt leading to another clutch.

Previous studies have demonstrated that males prefer to copulate with *Wolbachia*‐free females rather than with *Wolbachia*‐infected females when both are virgins (Moreau et al., 2001). However, male mate choice between virgin and mated females and how *Wolbachia* can affect this choice, as well as the existence of a refractory period in mated females’ long term after a first reproductive experiment, have never been investigated. However, in natural populations, males can interact with various females: virgin or experimented, *Wolbachia*‐infected, or *Wolbachia‐*free. We hypothesized that males can discriminate between virgin and mated females. We tested this hypothesis using experiments designed to determine the effects of a previous female reproductive experience and *Wolbachia* infection on sexual selection.

This study is the first in isopods to investigate male mate choice between virgin and experienced females, defined as females that had mated and produced offspring 6 months before the experiment in this context. Males could choose between females of these two types through two behavioral tests: (1) Using the open‐field choice test, we investigated male preference for virgin and experienced females and the behavioral reaction of females to these interactions (2) using the Y preference test, we compared the attractiveness of virgin and experienced females for males based on chemical cues. In order to compare the importance of reproductive status and infection status in male mate choice, these tests were performed with both *Wolbachia‐*free females and *Wolbachia*‐infected females. We also tested male preference for virgin *Wolbachia‐*infected females or experienced *Wolbachia‐*free females. In order to explain mate choice, we investigated some potential mechanisms involved, such as chemical profile and morphological characteristics.

## 2 METHODS

### 2.1 Animal rearing

Specimens of *Armadillidium vulgare* (Isopoda, Oniscidea, Latreille, 1804) were the descendants of *Wolbachia‐*free individuals collected in Helsingör (Denmark) in 1988, maintained at 20°C with the natural photoperiod in Poitiers (latitude 46°40′N), using controlled reproduction by selecting mates with no kin relationship to avoid inbreeding. To obtain *Wolbachia‐*infected females, some of these animals were injected with *Wolbachia* wVulC strain extract from ovaries and nerve chord of infected individuals in 1989 (see Juchault, Legrand & Martin 1974; Bouchon et al., 1998). *Wolbachia w*VulC strain is considered as the most geographically widespread in *A. vulgare,* and this strain has the highest transmission rate and virulence (Cordaux, Michel‐Salzat, Frelon‐Raimond, Rigaud, & Bouchon, 2004). The descendants of these females were then vertically infected with *Wolbachia* (hereafter “*Wolbachia*‐infected”). In infected lineages, all individuals that are genetically male and vertically infected are feminized by the bacteria *Wolbachia* (Cordaux et al., 2011). Males used for the breeding were the same for *Wolbachia‐*free females and *Wolbachia*‐infected females, in order to maintain the same genetic background. Ten *Wolbachia‐*free females and *Wolbachia*‐infected females were randomly selected and tested for *Wolbachia,* and PCR analysis confirms either the presence or the absence of the bacteria in the proper group (see Templé and Richard, 2015). The animals were all maintained in the same laboratory conditions. They were reared in boxes (26 × 13 cm) containing moistened soil with food (dead leaves and slices of fresh carrots) provided ad libitum. Males and all females were reared in separate boxes in order to avoid reproduction.

During their lifetime, individuals go through several molts with different physiological changes. Vitellogenesis is strongly correlated with the monthly molt cycle, and all female attractiveness increase until reaching a peak at the beginning of the pre‐ecdysis stage which lasts only 2–3 days (Beauché & Richard, 2013; Moreau & Rigaud, 2002). During ecdysis, when they lose their cuticle, both males and females are vulnerable and unable to mate (Moreau & Rigaud, 2000). In order to avoid attractiveness bias due to unsynchronized molting cycles, all the females used for the experiment were chosen at the beginning of the pre‐ecdysis stage, identified by the presence of white calcium carbonate shapes, previously described by Moreau and Rigaud (2002), about 5–10 days before ecdysis. Males were used during the intermolt stage, which corresponds to their preferential mating period (Moreau & Rigaud, 2000).

### 2.2 Experimental design

In this experiment, we compared the attractiveness and the behavior of virgin and experienced females. Virgin females had never encountered a male before the experiment (no mating experience). In contrast, experienced females had mated with a male, had one or two clutches, and were grouped for 6 months to rest before the experiment. For this experiment, all males and females observed were sexually mature adults aged 1.5 years.

Each virgin male was given the opportunity to interact with two females in three types of encounters: (1) a virgin and an experienced female, both of which were *Wolbachia‐*free, (2) a virgin and an experienced female, both of which were *Wolbachia*‐infected, and (3) a virgin *Wolbachia*‐infected female and an experienced *Wolbachia‐*free female. All observations were made in the same light and temperature conditions (10 Lux, 20°C). The cuticles of the male, virgin, and experienced females were marked with different colors the day before the behavioral experiment (with a Posca^®^ marker) to avoid residual odor.

### 2.3 Experiment 1: Open‐field choice test: behavioral interactions between individuals

**Figure 1:**
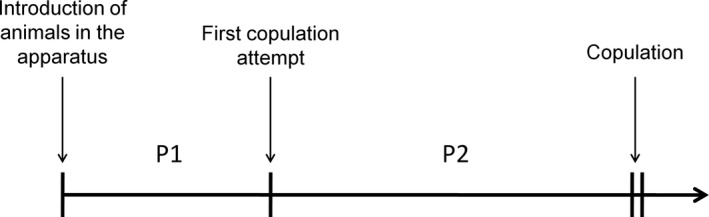
Schematic representation of the chronology of the open‐field test. Different items were recorded during the experiment. Between the introduction of the animals into the apparatus and the first copulation (P1), we recorded the occurrence and the duration of the male's interactions with the two types of females, and the type of female the first copulation attempt was aimed at. Between the first copulation attempt and the first copulation (P2), we recorded the number and the duration of copulation attempts with the two types of females and the females’ behavioral responses to the copulation attempts

In order to observe behavioral interactions between individuals, we performed an open‐field choice test, in a Petri dish (8 cm diameter), covered with moistened filter paper which was replaced after each experiment. Different behaviors were recorded during the experiment. During period 1 (P1 in Figure 1), which corresponds to the time of the introduction of the animals into the apparatus up until the first attempt at copulation, we recorded the occurrence and duration of the interactions initiated by the male (exploration of body with antennae) with the females. During period 2 (P2 in Figure 1), which corresponds to the duration from the first attempt at copulation up until the first copulation, we recorded the following:

- Copulation attempts (occurrence and duration) by the male with both females, in which the male explores the female body for a long time and mounts the female's dorsal surface.
- The female's reaction to the copulation attempts (described in Mead, 1973): rolling with opening (volvation followed by opening the body), rolling without opening, escape (moving away from the male), immobilization, toppling (jolting movements after the male's mount).
- Copulation: when a male performs copulation, characterized by a copulation attempt followed by volvation and the opening of the female body (described in Mead, 1973). The female chosen was also recorded.

The experiment ended after the first copulation. If no females accepted copulation, the experiment was stopped after 4 hr. A total of 19 replicates were carried out for the encounters with *Wolbachia‐*free virgin and experienced females*,* 17 replicates were performed for the encounters with *Wolbachia*‐infected virgin females and experienced females, and 16 replicates were conducted for the encounters with virgin *Wolbachia*‐infected females and experienced *Wolbachia‐*free females.

### 2.4 Experiment 2: Y preference test: attractiveness of virgin and experienced females from short distances

Previous studies conducted in our laboratory have shown that males prefer *Wolbachia‐*free females (Moreau et al., 2001; Richard, 2017). In order to ensure the same experimental conditions (period where females are attractive for males), we performed Y preference tests (*N* = 25 for each type of encounter) in which males could choose between two virgin females: one *Wolbachia‐*free and the other *Wolbachia*‐infected.

**Figure 2:**
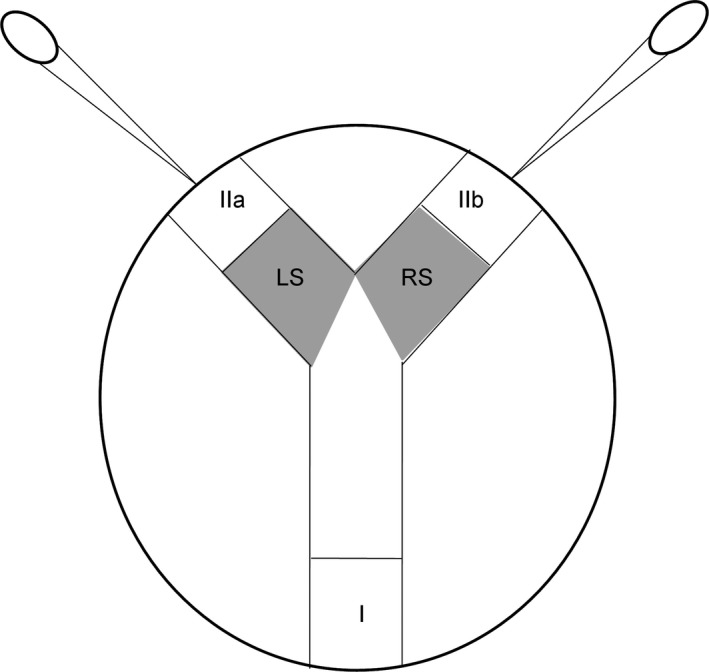
Schematic view of the Y preference test used to test the detection of females by males (I: initial position of tested male, IIa and IIb: positions of target females, LS: left side; RS: right side) with two plastic pipettes placed at the end of each tunnel and used to pulse air into the system at regular intervals. From Beauché and Richard (2013)

In order to test the females’ attractiveness, we used a Y‐shaped choice chamber (Figure 2, as set up by Beauché & Richard, 2013) built in a plastic Petri dish (9.5 cm diameter) covered with filter paper that was replaced after each experiment. The plastic parts of the device were cleaned with alcohol every day after the experiments. Rigid plastic tunnels were used to create the apparatus. In addition, two plastic pipettes were sealed at the end of these tunnels to pulse air regularly into the system passing through sections (IIa) and (IIb) to spread the odor of the animals presented for choice. Fifteen minutes before the experiment began one virgin and one experienced female was placed in sections (IIa) and (IIb) which are separated from the rest of the apparatus by mesh. Next, the male was placed in section (I) and released. The position of the target females in sections (IIa) and (IIb) was inverted after each experiment. Moreover, the target and tested individuals were each used only once. We recorded the time spent by the male in the left‐hand (LS) and the right‐hand sections (RS) connecting sections (IIa) and (IIb), respectively. The program EthoLog 2.2 (Ottoni, 2000) was used for the real‐time recording of movement. Each test began when the male moved from its initial position (I in Figure 2), and lasted for 10 minutes. In total, 25 replicates were carried out for all types of encounters. Males’ natural preference for one side (right or left) was not significant (Wilcoxon rank test: *Z* = 0.03, *N* = 75, *p* = .97).

### 2.5 Chemical analyses

We compared the chemical profile of *Wolbachia‐*free virgin females (*N* = 11), *Wolbachia*‐infected virgin females (*N* = 10), *Wolbachia‐*free experienced females (*N* = 13), and *Wolbachia*‐infected experienced females (*N* = 11). All females were sacrificed by freezing and stored at −20°C until extraction. In order to avoid contamination with internal body content, we verified that used females were not injured as cannibalism is common in this species. In our experiment condition, none of the individuals were injured. Individuals were immersed in 1 ml of dichloromethane for 24 hr. All samples remained completely transparent and were used for analysis. Before analysis, the samples were left to evaporate until dry and redissolved in 50 μl of dichloromethane.

Chemical profile analyses were conducted using gas chromatography FID. We analyzed 2 μl of solution using a 7890 GC‐FID (Agilent). A splitless injection was made into an Agilent capillary column DB‐5 (30 m × 0.250 mm, film = 0.50 μm) operated at 140°C for 2 min, increased by 5°C/min to 300°C, and kept at this temperature for 10 min. The injector and detector were kept at 300°C. The protocol is a modified version of the one used by Richard, Poulsen, Drijfhout, Jones, and Boomsma (2007).

### 2.6 Ethical treatment

Within the context of Directive 2010/63/EU on the protection of animals used for scientific purposes, the European Commission decided that most of invertebrate, including *A. vulgare*, crustacean isopoda, are excluded from ethical statement. However, we took numerous precautions during our study. Animals used were not collected in the field but were collected from our laboratory rearing. All individuals used for the study were raised in groups and were provided with food ad libitum. The behavioral experiments did not result in injury. After being used in a behavioral experiment, animals were maintained in groups in large boxes. Finally, the few animals used for chemical analysis and for microscopy were frozen.

### 2.7 Data analyses

The time spent by the males in the right and left sections of the Y preference test chamber, and the occurrence and duration of the copulation attempts with the virgin and the experienced females in the open‐field tests were compared using the nonparametric Wilcoxon test. The distribution of the different behavioral responses to the copulation attempts, and the proportion of females that accepted the copulation were compared between virgin and experienced females using Fisher's exact test. All of these tests were performed using the R software package (Team, R. D. C., 2008).

Chemical profiles were characterized by means of GC peak integration using the relative abundance of the various peaks. A stepwise discriminant analysis was performed to assess profile similarity, and the Mahalanobis distances (MD) between the centroids of the different groups were compared. The discriminant analysis was performed using Statistica 6.0 (Statsoft Inc.). The significance threshold was set at *p* ≤ .05.

## 3 RESULTS

### 3.1 Open‐field choice test: behavioral interactions between individuals

#### 3.1.1 Interactions before the first copulation attempt (P1)

Before the first copulation attempt, the number and the duration of the male's interactions did not significantly differ between the virgin and the experienced females for any of the three types of encounters (Wilcoxon signed‐ranks test: number and duration of interactions respectively, two *Wolbachia‐*free females: *Z* = 0.04, *N* = 19, *p = *.96; *Z* = 0.81, *N* = 19, *p = *.23; two *Wolbachia*‐infected females: *Z* = 1.46, *N* = 17, *p = *.37; *Z* = 1.46, *N* = 17, *p = *.14; a virgin *Wolbachia*‐infected female and an experienced *Wolbachia‐*free female: *Z* = 1.57, *N* = 17, *p = *.12; *Z* = 0.35, *N* = 16, *p = *.73; Table 1). Moreover, the males’ first copulation attempts were not preferentially directed toward either the virgin or the experienced females (Fisher's exact test, both females *Wolbachia‐*free: *N* = 19, *p = *.53; both females *Wolbachia*‐infected: *N* = 17, *p = *.49; virgin *Wolbachia*‐infected female and experienced *Wolbachia‐*free female: *N* = 16, *p = *1; Table 1).

**Table 1:** Summary of the open‐field observations before the first copulation attempt, for the three types of encounters: (1) virgin (V) and experienced (Exp) females both *Wolbachia‐*free (W−) (*N* = 19), (2) both *Wolbachia*‐infected (W+) (*N* = 17), and (3) virgin (V) *Wolbachia*‐infected (W+) females and experienced (Exp) *Wolbachia‐*free (W−) females (*N* = 16). The values observed did not significantly differ between virgin and experienced females

V W− vs.		Exp W−	V W+ vs.	Exp W+	V W+ vs.	Exp W−
First copulation attempt	8	11	10	7	8	8
Number of interactions by the male before the first copulation attempt (mean ± *SE*)	1.16 ± 0.26	1.16 ± 0.19	1.47 ± 0.29	1.12 ± 0.22	6.93 ± 1.5	5.64 ± 1.02
Duration (s) of male interaction before the first mount (mean ± *SE*)	14.26 ± 4.68	15.58 ± 5.13	21.94 ± 10.81	6.47 ± 4.87	75.98 ± 19.09	82.34 ± 18.11

#### 3.1.2 Copulation attempts

**Figure 3:**
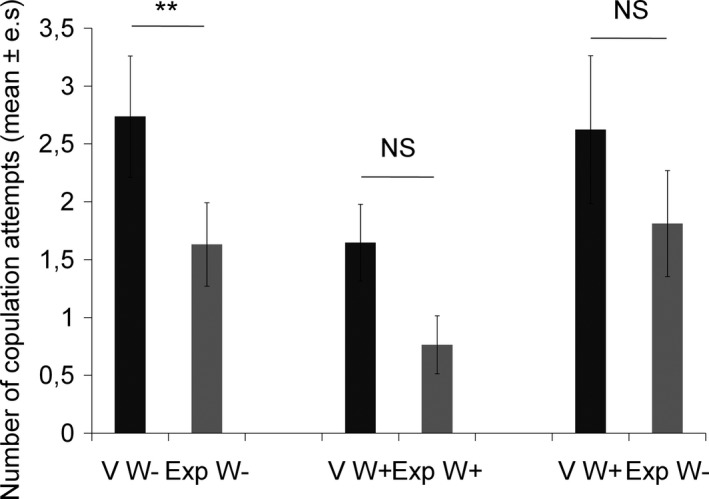
Number of copulation attempts from the beginning of the open‐field test until the first copulation for the three types of encounters: (1) virgin (V) and experienced (Exp) females both *Wolbachia‐*free (W−) (*N* = 19), (2) both *Wolbachia*‐infected (W+) (*N* = 17), and (3) virgin *Wolbachia*‐infected females and experienced *Wolbachia‐*free females (respectively, W+ and W−, *N* = 16). Wilcoxon signed‐ranks test: *p ≤ .05; **p ≤ .01; NS: *p* > .05

Males made significantly more attempts at copulation with virgin females than with experienced females when both were *Wolbachia‐*free (Wilcoxon signed‐ranks test: *Z* = 2.79, *N* = 19, *p = *.005; Figure 3). There was a trend toward more copulation attempts with virgin females than with experienced females when they were both *Wolbachia*‐infected, but the difference was not significant (Wilcoxon signed‐ranks test: *Z* = 1.88, *N* = 17, *p = *.06; Figure 3). However, the number of copulation attempts made by the male did not differ between virgin *Wolbachia*‐infected females and experienced *Wolbachia‐*free females (Wilcoxon signed‐ranks test: *Z* = 1.08, *N* = 16, *p = *.27; Figure 3).

**Figure 4:**
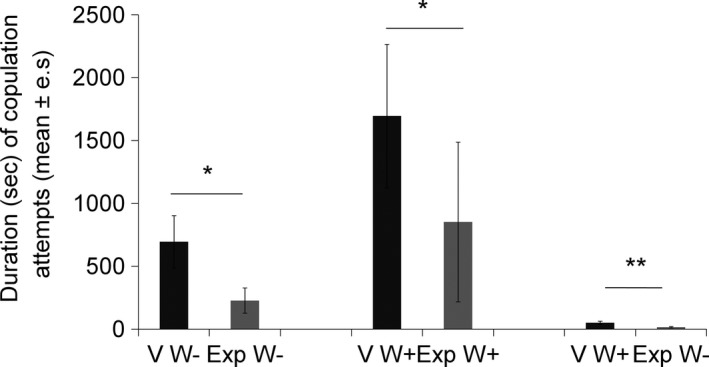
Duration of copulation attempts from the beginning of the open‐field test until the first copulation, for the three types of encounters: (1) virgin (V) and experienced (Exp) females both *Wolbachia‐*free (W−) (*N* = 19), (2) both *Wolbachia*‐infected (W+) (*N* = 17), and (3) virgin *Wolbachia*‐infected females and experienced *Wolbachia‐*free females (respectively, W+ and W−) (*N* = 16). Wilcoxon signed‐ranks test: *p ≤ .05; **p ≤ .01; NS:* p* > .05

Copulation attempts were longer with virgin than with experienced females for the three types of encounters (both females *Wolbachia‐*free: *Z* = 1.93, *p = *.05; both females *Wolbachia*‐infected: *Z* = 1.96, *p = *.04; virgin *Wolbachia*‐infected female and experienced *Wolbachia‐*free female: *Z* = 2.53, *p = *.01; Figure 4).

#### 3.1.3 Behavioral responses to copulation attempts

When both females were *Wolbachia‐*free, the proportion of the different responses to copulation attempts differed between virgin and experienced females (Fisher's exact test, *p = *.02, Table 2). For the other combinations, the proportion of each behavioral response did not significantly differ between the females according to their reproductive status (Fisher's exact test, respectively, *p = *.21 and *p = *.83, Table 2).

**Table 2:** Occurrence of the different behavioral responses to copulation attempts during the open‐field test with virgin and experienced females, according to their infection status (W−: *Wolbachia‐*free; W+: *Wolbachia*‐infected)

	V W − vs. Exp W−		V W+ vs. Exp W+		V W+ vs. Exp W−	
Rolling and opening	9	0	10	2	0	0
Rolling without opening	6	6	5	5	0	0
Immobilization	34	28	15	11	24	19
Escape	8	2	2	3	16	9
Topple	2	3	0	1	2	1

#### 3.1.4 Copulations

More virgin females than experienced ones accepted copulation, regardless of whether they were *Wolbachia*‐infected or *Wolbachia‐*free (Table 3). However, when the male was able to choose between a virgin *Wolbachia*‐infected and an experienced *Wolbachia‐*free female, no copulation was observed (Table 3).

**Table 3:** Number of virgin (V) and experienced (Exp) females that accepted and refused copulation in each experiment, according to their infection status (W−: *Wolbachia‐*free; W+: *Wolbachia*‐infected) and significance of the Fisher's exact test

	V W− vs. Exp W−		V W+ vs. Exp W+		V W+ vs. Exp W−	
Number of females which accepted the copulation	8	0	9	1	0	0
Number of females which refused the copulation	11	19	8	16	16	16
Significance of the Fisher's exact test	***p*** ** = .003**		***p*** ** = .006**		***p*** ** = 1**	

### 3.2 Y preference test: the attractiveness of virgin and experienced females at short distances

**Figure 5:**
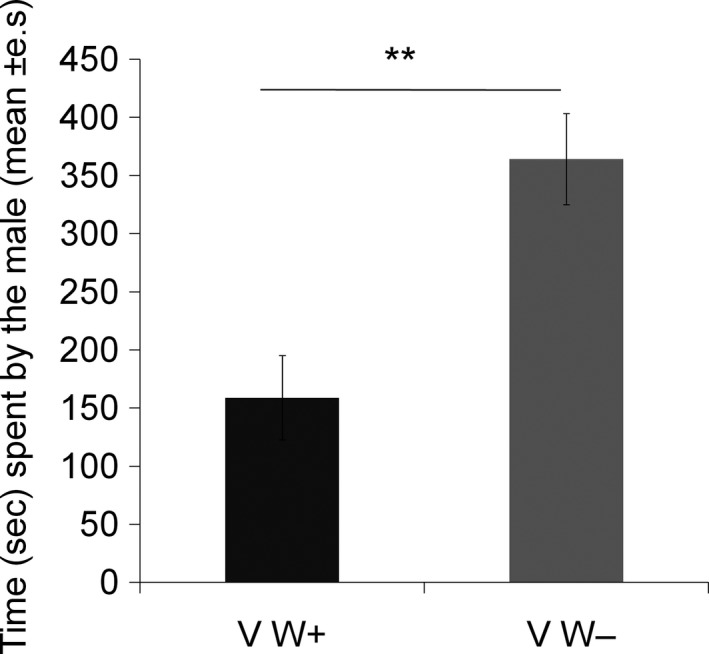
Time spent by males in the right‐hand (RS) and left‐hand (LS) sections occupied by *Wolbachia*‐infected (V W+, *N* = 25) females or *Wolbachia‐*free (V W−, *N* = 25) females, both virgins. Wilcoxon signed‐ranks test: **p ≤* *.01

In the setup with two virgin females, the males spent significantly more time in front of the section adjacent to the *Wolbachia‐*free female than the section adjacent to the *Wolbachia*‐infected female (Wilcoxon signed‐ranks test: *Z* = 2.32, *N* = 25, *p = *.01; Figure 5).

**Figure 6:**
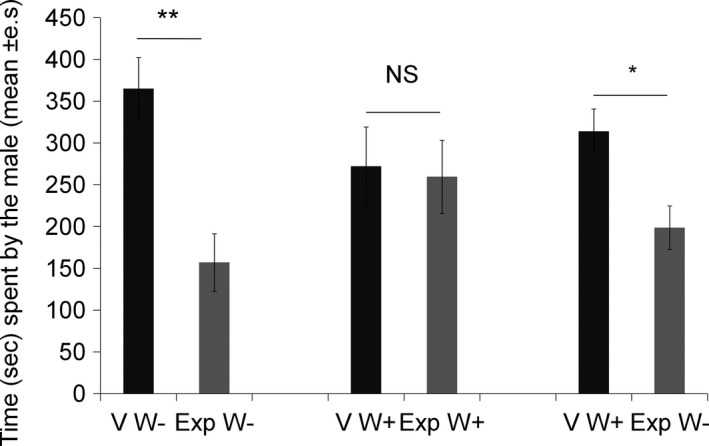
Time spent by males in the right‐hand (RS) and left‐hand (LS) sections occupied by virgin (V) or experienced (Exp) females, for the three types of encounters: (1) virgin (V) and experienced (Exp) females both *Wolbachia‐*free (W−) (*N* = 25), (2) both *Wolbachia*‐infected (W+) (*N* = 25), and (3) virgin (V) *Wolbachia*‐infected (W+) females and experienced (Exp) *Wolbachia‐*free (W−) females (*N* = 25). Wilcoxon signed‐ranks test: **p ≤* *.01; *p ≤* *.05; NS:* p *>* *.05

When the females were both *Wolbachia‐*free, the males spent significantly more time in front of the section adjacent to the virgin females than in front of the section adjacent to the experienced females (Wilcoxon signed‐ranks test: *Z* = 2.5, *N* = 25, *p = *.01; Figure 6). However, male preference did not significantly differ between females when both were *Wolbachia*‐infected (Wilcoxon signed‐ranks test: *Z* = 0.18, *N* = 25, *p = *.85; Figure 6). When the virgin females were *Wolbachia*‐infected, and the experienced females were *Wolbachia‐*free, the males spent significantly more time in front of the section adjacent to the virgin females (Wilcoxon signed‐ranks test: *Z* = 2.2, *N* = 25, *p = *.02; Figure 6).

## 4 CHEMICAL ANALYSIS

**Figure 7:**
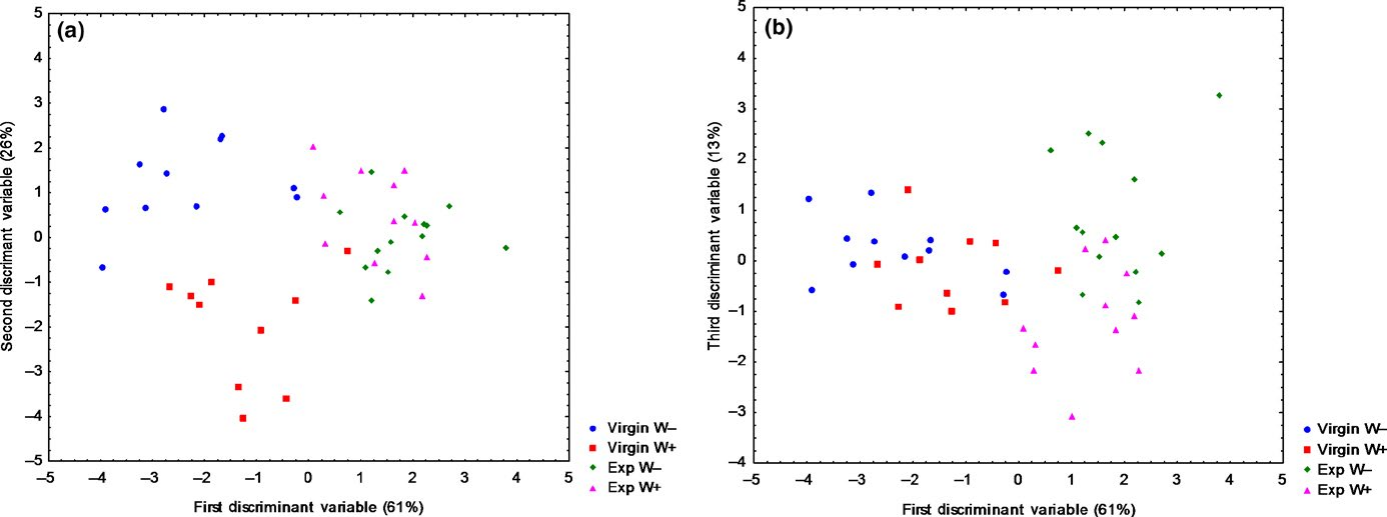
(a, b) Chemical profile analyses of virgin females, *Wolbachia‐*free (Virgin W−) and *Wolbachia*‐infected (Virgin W+), and experienced females, *Wolbachia‐*free (Exp W−) and *Wolbachia*‐infected (Exp W+) (N between 10 and 13 for each group). A discriminant analysis was performed on the chemical profiles based on the relative proportions of chemical compounds [*F*(30, 94) = 5.25; *p* < .0001]. Data were obtained from gas chromatography analysis of chemical extracts

The analysis of the relative proportion of peaks observed on the gas chromatograph from chemical extracts from the different female groups of *A. vulgare* revealed three variables (Variable 1: 61%, Variable 2: 26%, and Variable 3: 13%; Figure 7a,b). Females presented variations in the relative proportion of chemical compounds according to their mating experience and also according to the presence of *Wolbachia* [*F*(30, 94) = 5.25; *p* < .0001]. The Mahalanobis distances (MD) between the group centroids of the different groups were significant overall (All MD, *p* < .05). Chemical quantities were too low for proper identification but when possible the compound family was identified by mass spectrometry (GC‐MS). We then mainly found hydrocarbons, ketones, and fatty acids. Moreover, no phthalate pollution was in our extracts.

## 5 DISCUSSION

Our results revealed that males are more attracted to virgin females compared to experienced females in the terrestrial isopod *Armadillidium vulgare*. Males were able to discriminate between and show a preference for females from short distances based on their reproductive status. This preference also correlates with the differing blends of chemical compounds found in virgin and experienced females. Moreover, when individuals could interact directly, males attempted to copulate more frequently and for longer with virgin females while most experienced females refused further copulation. Interestingly, when the experienced and the virgin females were both *Wolbachia*‐infected, the male did not choose from short distances based solely on odor but expressed different levels of mating interest between both females when allowed to interact. Finally, in the open‐field choice test, males preferred virgin females even if they were *Wolbachia*‐infected*,* suggesting that males discriminate based on female reproductive status and this criterion is used for male mate choice.

### 5.1 Experienced females are less attractive than virgin females

Behavioral tests indicate that reproductive experience actively induces a reduction in the attractiveness of experienced females, and demonstrate the male's capacity to discriminate between the two types of females from short distances, based on their odor and by contact.

A postmating decrease in attractiveness due to chemical changes has been demonstrated in other arthropods, but in most studies, the females were tested only a few hours postmating, and the influence on producing offspring was not investigated (Carazo et al., 2004; Durgin, Martin, Watkins, & Mathews, 2008; Schneider et al., 2016). In the beetle *Tenebrio molitor*, males can assess the mating status of females with chemicals cues present on filter paper on which a virgin or a mated female was placed for a 24‐hour period before the trial (Carazo et al., 2004). In this study, males were more attracted to the odor of the virgin female than to the odor of the mated female, placed with a male just before the experiment (Carazo et al., 2004).

The reasons for this change in odor may differ between species. In some species, males deposit an anti‐aphrodisiac pheromone on the female's body during copulation, and their seminal fluid may also contain compounds responsible for the decrease in female attractiveness (e.g., in *Drosophila melanogaster*: Scott, 1986; Scott, Richmond, & Carlson, 1988; Tram & Wolfner, 1998; in the butterfly *Heliconius melpomene*: Schulz, Estrada, Yildizhan, Boppré, & Gilbert, 2008). In addition, mated females may secrete aversive chemical cues that decrease attractiveness, as with *D. melanogaster* (Scott, 1986) and with the parasitoid wasp *Spalangia endius* in which females emit this anti‐aphrodisiac pheromone when they are courted by new males (King et al., 2005; Mowles, King, Linforth, & Hardy, 2013).

In our study, females were mated at least 6 months before the choice test and in the interim had no contact with males. *A. vulgare* is a species which molts every month, so the decrease in the attractiveness of experienced females is not the result of male odor. However, during the 6 months postmating, experienced females stored male sperm in their spermatheca and had one or two clutches, which could also lead to a change in their odor. When males could interact with females during the open‐field test, they made more attempts at copulation, and these were longer when they were directed toward the virgin females. A previous study of *A. vulgare* showed that males were attracted to females regardless of their reproductive status (virgin, just mated or 2 months after a previous mating, Moreau et al., 2002), but individuals were tested in pairs (one male with only one female) in which males could not choose between two females. Concerning the male behavior when they could interact with females, author did not report any “delays” in males approaching experienced females.

Multiple matings lead to spermatic competition. Being the last mated male offers benefits in terms of paternity (Mestre, Rodríguez‐Teijeiro, & Tuni, 2015; Simmons, 2001; Siva‐Jothy & Tsubaki, 1994; Zeh & Zeh, 1994). However, in terrestrial isopods, studies have shown no sperm precedence from consecutive matings with different males (Johnson, 1982; Moreau et al., 2002), and the last male shares paternity with previous partners. The acquisition of strategies to recognize and avoid courting mated females decreases the probability of sharing paternity.

Moreover, the experienced females used in our study were submitted to previous mating and to offspring production, in contrast with most studies on the behavioral and physiological impact of mating. Although mating may be costly (see below), for example, egg production may also have a cost in terms of survival (e.g., in *Ceratitis capitata*, Chapman, Takahisa, Smith, & Partridge, 1998). Such costs may exist in *A. vulgare*, as females store their eggs in a marsupium for 1 month, until the birth of their offspring. Indeed, parental care may induce energetic and survival costs, for example, by increasing the calorific expense or by decreasing escape behavior (Gilbert, Thomas, & Manica, 2010). Therefore, we can hypothesize that experienced females have less chance of carrying their future offspring to term than virgin females.

### 5.2 Experienced females refused new copulation

Our results revealed that experienced females are more resistant to the copulation attempts. Indeed, experienced females, when they were courted by males, never accepted the copulation: They did not demonstrate any “rolling and opening” behavior during the male's copulation attempts.

A previous study revealed that, in *A. vulgare*, mated females were less receptive to further copulation, but an average of 25% of females accepted to mate again just a few minutes postmating and 90% of females were willing to mate again 2 months after their previous mating and after being isolated from males (i.e., 1 month after they have produced offspring) (Moreau et al., 2002). These results suggest the absence of a complete refractory period. However, our results revealed that 6 months postmating and after the birth of their offspring, females refused new copulation. This behavioral resistance has already been observed in other species. Female snails (*Neptunea arthritica)* increased their resistance with successive matings (Carlos & Seiji, 2011), and for the ladybird *Adalia bipunctata*, remating was more frequently observed with large males, better able to overcome resistance (Perry, Sharpe, & Rowe, 2009). This behavioral resistance, costly for the female, suggests that refusing to mate could be more beneficial for experienced females than accepting. Indeed, in species without nuptial gifts, mating may have negative effects, on survival, for example (Arnqvist & Nilsson, 2000). In some species, a decrease in survival after mating is induced by the male copulatory organ (Blanckenhorn et al., 2002; Crudgington & Siva‐Jothy, 2000; Shine et al., 2004). During copulation, male *A. vulgare* insert their endopodite 1 and 2, which come together to form a gutter to transfer sperm, into the female genitalia opening. The intromission of these endopodites, because of their size and rigidity, can injure the females. Moreover, the results obtained from SEM observation (see Appendix) indicate that a part of endopodite 1 has spiny structures the function of which remains unknown. In the bean weevil *Callosobruchus maculates* and in the dung fly *Sepsis cynipsea*, the male genitalia harbor numerous spiny chitinous structures which damage the female genitalia (Blanckenhorn et al., 2002; Crudgington & Siva‐Jothy, 2000). For these females, mortality increases after the first mating, and in particular after a second mating. There may also be a cost relating to compounds in the seminal fluid received by the female during mating, which have several effects, including reducing life span and defense against infections (Chapman, Liddle, Kalb, Wolfner, & Partridge, 1995; Lung et al., 2002; Short, Wolfner, & Lazzaro, 2012). In *A. vulgare*, none of these costs have yet been discovered, and the causes of mating costs cannot yet be described. Scanning electron microscopy has allowed us to observe different structures of the male reproductive organ. Moreover, comparison of virgin and experienced females’ genital apertures revealed no visible differences on SEM images (Appendix). These new data give us a better knowledge of *A. vulgare* reproduction and pave the way for further studies requiring other specialities.

### 5.3 The Wolbachia infection modulates these preferences, but seems to be less important than reproductive status

When both females were virgin, Y preference tests showed that *A. vulgare* males spent more time near the *Wolbachia‐*free females than the *Wolbachia*‐infected females. This finding supports results obtained for the same species in open‐field test (Moreau et al., 2001). In this previous study, *A. vulgare* males perform more insemination events with uninfected females (Moreau et al., 2001). In the present study, the chemical analysis showed that the chemical profile of females differed according the presence or the absence of *Wolbachia*, allowing the males to discriminate based on odor cues. The mechanism behind this discrimination is not yet known, and several hypotheses may explain this discrimination. For example, in *Drosophila*, commensal bacteria altered the profile of cuticular hydrocarbons and influence mate choice (Ringo, Sharon, & Segal, 2011; Sharon et al., 2010). In *A. vulgare*, one probable explanation is that the *Wolbachia* bacteria alter the synthesis of cuticular compounds. Indeed, an important site of infection was found in the rosette glands, which may be involved in synthesizing the cuticle at each molt (Gorvett, 1946).

An ability to discriminate based on infection status in a mate choice context can be advantageous if the infection is costly for its host. In terrestrial isopods, *Wolbachia* decrease the fitness of the host and have a negative effect on the immune system (Braquart‐Varnier et al., 2008), growth (Juchault & Mocquard, 1989), fertility (Moreau et al., 2002), and the number of descendants (Fortin, Souty‐Grosset, & Richard. The choice of uninfected individuals as mates is common in several species (Penn, Schneider, White, Slev, & Potts, 1998; Worden & Parker, 2001, 2005; Worden, Parker, & Pappas, 2000). In *Tenebrio molitor*, females are more attracted to males not infected with *Hymenolepsis diminuta*, which leads to the production of more offspring (Worden & Parker, 2005; Worden et al., 2000). In contrast, in case of cytoplasmic incompatibility in which *Wolbachia* have mutualistic interactions with their host, the males do not choose or discriminate in favor of *Wolbachia‐*free females. Males usually are more likely to mate with females harboring the same strain of *Wolbachia* (Miller, Ehrman, & Schneider, 2010;  Ming, Shen, Cheng, Liu, & Feng, 2015).

When both females are *Wolbachia*‐infected, males do not show a preference for virgin or mated females in the Y preference test. However, in the open‐field tests, males perform more and longer copulation attempts with virgin females than with experienced females, even if they are both *Wolbachia*‐infected. This indicates that *Wolbachia*‐infected females are more attractive when they are virgin. It is possible that the difference in chemical cues between virgin and experienced females was too subtle to be detected in the Y preference test, perhaps because the *Wolbachia* signal was more specific, and males have to interact directly with the females to fully determine their reproductive experience. Uninfected individuals behave specifically with individuals infected by *Wolbachia*. We hypothesized that behavioral changes are probably due to specific pattern. Our results highlight that the odor could be one reason but we cannot exclude other phenotypes. Alternatively, this difference in courtship may be due to the female's behavior. When they are experienced, both females infected and uninfected with *Wolbachia* were more resistant to mating, revealing that the infection does not decrease the costs associated with a second mating. Moreover, comparative observations of genital apertures between *Wolbachia‐*free vs. *Wolbachia*‐infected females using SEM images showed no morphological differences. (Appendix). Such comparisons of morphological aspects between these two kinds of females were investigated for the first time.

To conclude, this study is the first to investigate the long‐term effects of previous mating and of producing offspring on female attractiveness. These previous experiences cause differences in the females’ chemical profile, detected by males at short distances. Indeed, unlike in insects, the compounds present in the cuticle of *A. vulgare* are in very low quantity, and so their full identification remains complex. However, mass spectrometry identification was used to remove all contaminants from the statistical analyses.

The cause of the difference in attractiveness to males between virgin and experienced females is not yet known and should be investigated further. Sperm compound cues or active secretion by the female of an unattractive odor seems to be the most likely hypotheses.

For males, it seems to be costly to spend time and energy attempting to copulate with mated females because the females would be more resistant to mating, weaker, and not necessarily able to carry a new clutch. Moreover, if the males are successful, paternity may be shared. Experienced females are more resistant to copulation attempts, probably in order to avoid the costs associated with a second mating. *Wolbachia* infection leads to a decrease in male mate preference based on short distance cues but does not influence the impact of previous matings on female resistance to further mating, suggesting that the infection does not influence the cost of mating in this species.

## CONFLICT OF INTEREST

None declared.

## AUTHOR CONTRIBUTION

MF, CSG, and FJR contributed substantially to the experimental design. MF performed the behavioral experiments and acquired the SEM data. CD contributed to the experimental design and performed the chemical analyses. MF drafted the manuscript, and all of the coauthors revised it critically and approved this version.

## APPENDIX 1

### SCANNING ELECTRON MICROSCOPY

#### METHODS

The posterior part of two individuals (one male and one female) and the endopodite 1 and endopodite 2 of two other males were fixed in a solution of glutaraldehyde 9%, cacodylate 0.3 M, and NaCl 3% at pH 7.3, for 2 hr at 4°C. Specimens were postfixed in osmium tetroxide 4%; cacodylate 0.3 M, and NaCL 5.5%. Dehydration was conducted in a series of acetone (2 × 1 mn in 50% acetone, 2 × 5 mn in acetone 70, 2 × 15 mn in acetone 90, and 4 × 15 mn in acetone 100). Samples were then critical‐point dried with a CDP 030 BALZERS and embedded in platinum. The samples were viewed using a Jeol 5600 LV JSM at 20 kV using an Ultrascan 894 GATAN digital camera (512 × 512 pixels) and Microphoton LC micro software.

#### OBSERVATIONS

The male copulatory organ structures are comprised of one endopodite 1 (En 1) and one endopodite 2 on each side, which form a gutter which enters into the female's genital opening during copulation. A ventral view of the male copulatory organ shows the two endopodites 1 at the base of the exopodite 1 (Ex 1), the uropode (U in Appendix, A_1_). During copulation, the endopodites 1 (Appendix, A_2_) and 2 (En 2 in Appendix, A_3_) on the same side join together to form a gutter which conducts sperm. Long silk strand is present along andopodite 1 (S1 in Appendix, A2). Endopodite 1 is approximately 100 μm wide and 1.6 mm long. In A_2_, long strands of silk can be seen alongside the endopodite 1. At the distal extremity of the endopodite 1, we can observe two structures, the role of which is unknown: in A_5_, short strands of silk appear at the top of the distal extremity of the endopodite 1 (S2); in A_6_, a row of serrated structures can be seen at the end of the endopodite 1. Endopodite 2 is more flexible and thinner than endopodite 1 and is approximately 40 μm wide and 2 mm long. In females, both genital openings (right and left) are on the internal side of the 5th pair of legs (GO in Appendix, B_0_). The genital openings have a diameter of around 80 μm, for the *Wolbachia‐*free females, virgin (B_1_) and experienced (B_2_) and for the *Wolbachia*‐infected females virgin (B_3_) and experienced (B_4_) (Appendix). We did not notice any differences between the different kind of females, according their infectious and reproductive status.
